# CAR T-Cell Persistence Correlates with Improved Outcome in Patients with B-Cell Lymphoma

**DOI:** 10.3390/ijms24065688

**Published:** 2023-03-16

**Authors:** Valerie Wittibschlager, Ulrike Bacher, Katja Seipel, Naomi Porret, Gertrud Wiedemann, Claudia Haslebacher, Michèle Hoffmann, Michael Daskalakis, Dilara Akhoundova, Thomas Pabst

**Affiliations:** 1 Department of Medical Oncology, Inselspital, University Hospital of Bern, 3010 Bern, Switzerland; 2 Department of Hematology and Central Hematology Laboratory, Inselspital, Bern University Hospital, University of Bern, 3010 Bern, Switzerland; 3 Center of Hemato-Oncology, University Cancer Center, 3010 Bern, Switzerland

**Keywords:** CAR T-cell persistence, CAR T-cell therapy, B-cell lymphoma, ddPCR

## Abstract

Chimeric antigen receptor (CAR) T-cell therapy has led to profound and durable tumor responses in a relevant subset of patients with relapsed/refractory (r/r) B-cell lymphomas. Still, some patients show insufficient benefit or relapse after CAR T-cell therapy. We performed a retrospective study to investigate the correlation between CAR T-cell persistence in the peripheral blood (PB) at 6 months, assessed by droplet digital PCR (ddPCR), with CAR T-cell treatment outcome. 92 patients with r/r B-cell lymphomas were treated with CD19-targeting CAR T-cell therapies at our institution between 01/2019–08/2022. Six months post-treatment, 15 (16%) patients had no detectable circulating CAR-T constructs by ddPCR. Patients with CAR T-cell persistence had a significantly higher CAR T-cell peak (5432 vs. 620 copies/ug cfDNA, *p* = 0.0096), as well as higher incidence of immune effector cell-associated neurotoxicity syndrome (37% vs. 7%, *p* = 0.0182). After a median follow-up of 8.5 months, 31 (34%) patients relapsed. Lymphoma relapses were less frequent among patients with CAR T-cell persistence (29% vs. 60%, *p* = 0.0336), and CAR T-cell persistence in the PB at 6 months was associated with longer progression-free survival (PFS) (HR 2.79, 95% CI: 1.09–7.11, *p* = 0.0319). Moreover, we observed a trend towards improved overall survival (OS) (HR 1.99, 95% CI: 0.68–5.82, *p* = 0.2092) for these patients. In our cohort of 92 B-cell lymphomas, CAR T-cell persistence at 6 months was associated with lower relapse rates and longer PFS. Moreover, our data confirm that 4-1BB-CAR T-cells have a longer persistence as compared to CD-28-based CAR T-cells.

## 1. Introduction

B-cell lymphomas exhibit typically high sensitivity to chemoimmunotherapy, hence treatment usually leads to excellent tumor responses [1,2,3]. However, a fraction of patients still insufficiently respond or relapse, developing refractory lethal disease [1,2,4]. First-line consolidation with high-dose chemotherapy may improve long-term outcome in a subset of high-risk patients, but does not prevent relapses in all patients [1,5]. For patients with relapsed/refractory (r/r) disease, the development and approval of CD19-targeting chimeric antigen receptor (CAR) T-cell therapies has supposed a revolutionary treatment opportunity [1,2,6]. CAR T-cell therapies rely on genetic engineering of patient-derived T-cells [7,8,9]. These T-cells are collected from peripheral blood (PB), and in vitro modified to successfully bind specific tumor cell surface antigens, triggering potent antitumor responses [7,9]. In vivo, tumor antigen recognition leads to CAR T-cell activation and proliferation, with a proportion of CAR T-cells persisting long-term [10,11,12]. For treatment of B-cell malignancies, currently approved and commercially available CAR T-cell products target the B-cell surface antigen CD19 [13,14]. Three CAR T-cell products, tisagenlecleucel (Kymriah^®^), axicabtagene ciloleucel (Yescarta^®^) and lisocabtagene maraleucel (Breyanzi^®^), are FDA approved for patients with diffuse large B-cell lymphoma (DLBCL), r/r after two or more systemic treatment lines [2,7,8,15]. Tisagenlecleucel is also authorized for patients under 25 years diagnosed with r/r B-cell acute lymphoblastic leukemia (B-ALL) [7]. Brexucabtagen autoleucel (Tecartus^®^) is approved for patients with r/r mantle cell lymphoma (MCL) and follicular lymphoma (FL) [1]. These therapies have lead to unprecedentedly high rates of tumor responses and durable disease remissions. For instance, axicabtagene ciloleucel showed in the ZUMA-1 trial an objective response rate as high as 82%, with a complete response rate of 54%, in patients with r/r DLBCL, transformed FL and primary mediastinal B-cell lymphoma [8,13,16]. Furthermore, CAR T-cell therapy has shown outstanding success in patients with B-ALL, with complete response rates between 70% and 94% across distinct trials [13]. However, despite high rate of durable complete responses, some patients still relapse, regardless of the initial response. Underlying resistance and escape mechanisms are still incompletely understood, and investigation of these mechanisms constitutes an active research field [4,17]. Most frequent resistance mechanisms are related to insufficient CAR T-cell expansion and persistence, or due to tumor cell modifications (e.g., loss of the target antigen) [4,17]. For instance, CD19-positive B-ALL can relapse as CD19-negative disease as result of CAR T-cell selective pressure [6,18]. Therefore, further translational research is essential to optimize clinical outcomes of CAR T-cell therapy [19].

One attempt to better understand the heterogeneity in tumor responses is to analyse individual-patient CAR T-cell kinetics. As CAR T-cell therapies are replicating cell-based products, their pharmacokinetics relevantly differ from conventional drugs [20]. CAR T-cell kinetics can be monitored by polymerase chain reaction (PCR), which enables transgene quantification, or by flow cytometry, through identification of surface epitopes on live CAR T-cells [18]. Droplet digital PCR (ddPCR) is a new diagnostic approach that enables monitoring of CAR T-cell kinetics, with increasing application in translational research and clinical routine [6,19,21,22]. Multiple studies confirmed high reproducibility and precision of ddPCR measurements in replicate tests [6,19,23]. Following re-infusion, CAR T-cell kinetics are typically characterized by expansion, contraction and persistence phases [18]. However, there is high interpatient variability regarding magnitude and duration of these phases, which has been related to distinct product- and patient-specific factors [18]. Some previous studies have shown that a low-magnitude CAR T-cell expansion more frequently leads to therapeutic failure, and higher expansion correlates with increased incidence of side effects [11,19,24]. Furthermore, long-term persistence of CAR T-cells has been reported to be predictive of durable remissions [11,12,17,19,25]. Other studies could not demonstrate clear correlation between CAR T-cell kinetics and tumor responses [26]. Globally, given the relatively short follow-up in available CAR-T studies and lack of universal access to CAR T-cell monitoring tools, limited knowledge on CAR T-cell kinetics and correlation with treatment outcomes is available to date [24,27]. Within this study, we retrospectively analysed the impact of peripheral CAR T-cell persistence at 6 months on treatment outcome, including progression-free survival (PFS) and overall survival (OS), in a cohort of 92 patients with B-cell lymphomas. We used 6 months cut-off to segregate patients into two cohorts: patients with (n = 77, 84%) vs. without (n = 15, 16%) CAR T-cell persistence in the PB. Moerover, we performed a second stratification based on the CAR T-cell product received (Kymriah^®^ vs. Yescarta^®^/Tecartus^®^).

## 2. Results

### 2.1. Patient Baseline Characteristics

27 (29%) patients were female and 65 (61%) male. Median age at diagnosis was 62 years (range: 36–80). Most patients (85%) had a DLBCL histology. 57% of patients had a stage III-IV disease according to the Ann-Arbor staging system. Baseline characteristics were balanced between both patient cohorts. Stage III was more frequent (33% vs. 5%, *p* = 0.0051) in patients with CAR T-cell persistence, whereas no significant difference was observed for stage IV distribution. Prognostic scores and previous history of hematopoietic stem cell transplantation showed no differences. Patients’ baseline characteristics are summarized in Table 1.

### 2.2. Disease Features and CAR T-Cell Treatment

62 (67%) patients received 3 or more treatment lines previous to CAR T-cell therapy and 29 (32%) had undergone previous radiotherapy. Disease remission status previous to CAR T-cell therapy was most frequently progressive disease (PD) (49%) and partial response (PR) (37%). Bridging therapy was required in 47 (51%) patients. Median lactate dehydrogenase (LDH) level before start of lymphodepleting chemotherapy was 344 U/L (range: 126–3949). Median time between lymphapheresis and CAR T-cell therapy was 48 days (range: 27–221) and median duration of hospitalization was 21.5 days (range: 5–73). 55 (60%) patients received Kymriah^®^, 25 (27%) were treated with Yescarta^®^, and 12 (13%) received Tecartus^®^. Only 2 out of the 55 (13%) patients who received Kymriah^®^ had a negative CAR T-cell ddPCR within the first 6 months. Within the subgroup of patients treated with Yescarta^®^, 11 (73%) patients had undetectable CAR T-cell constructs in the PB 6 months post-treatment (*p* = < 0.0001). (Table 2).

While CRS and high-grade CRS frequency was similar between patients with and without CAR T-cell persistence (79% vs. 80%, *p* > 0.9999), immune effector cell-associated neurotoxicity syndrome (ICANS) occurred more frequently in patients with persistent CAR T-cells at 6 months (37% vs. 7%, *p* = 0.0182). 59 (64%) patients required tocilizumab and 48 (52%) steroids (Table 2, Supplementary Table S2).

### 2.3. CAR T-Cell Kinetics

**Figure 1:**
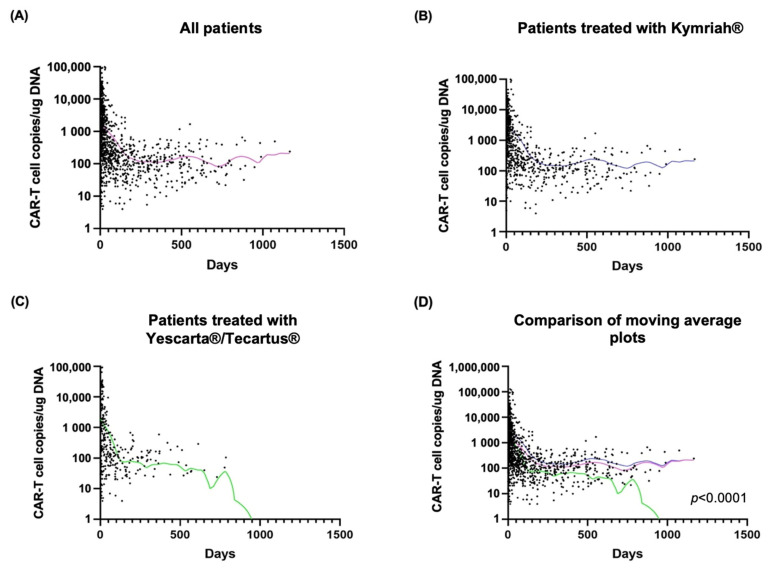
CAR T-cell dynamics in the PB in patients included in the study: (**A**) in the entire patient cohort; (**B**) in patients treated with Kymriah^®^; (**C**) in patients treated with Yescarta^®^/Tecartus^®^; (**D**) Comparison of moving average plots from A, B and C. Violet: average line for all patients; blue: average line for patients treated with Kymriah^®^; green: average line for patients treated with Yescarta^®^/Tecartus^®^.

After CAR T-cell infusion, a rapid expansion of CAR T-cells was observed, followed by a contraction phase characterized by a progressive decline in the circulating CAR T-cell count. Finally, while a proportion of patients showed stabilization of circulating CAR T-cell levels, in other patients a rapid decline occurred leading to undetectable levels. The median CAR T peak level was 4859.5 copies/ug cell-free DNA (cfDNA) for the whole study cohort, and was significantly higher in patients with CAR T-cell persistence (5432 vs. 620 copies/ug cfDNA, *p* = 0.0096). A similar difference was observed in the subgroup of patients receiveing Yescarta^®^/Tecartus^®^ (8297 vs. 620 copies/ug DNA, *p* = 0.0061). Median time to CAR T peak was 9 days (range: 2–83), with no significant differences between the cohort of patients with vs. without CAR T-cell persistence. 16% of patients showed no evidence of CAR T-cell persistence at 6 months post-CAR T-cell therapy. Median time to undetectable CAR T-cell constructs was 98 days (range: 17–651), and median time between CAR T-cell peak level and ddPCR negativization was 88 days (range: 10–642) (Figure 1, Table 3).

**Figure 2:**
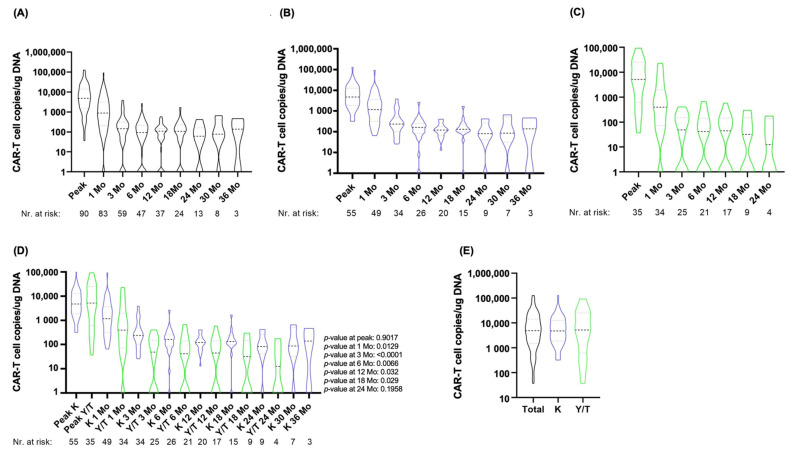
CAR-T cell construct concentrations in the PB at 1, 3, 6, 12, 18, 24, 30 and 36 months post-treatment. (**A**) in the entire patient cohort; (**B**) in patients treated with Kymriah^®^; (**C**) in patients treated with Yescarta^®^/Tecartus^®^; (**D**) comparison between the Kymriah^®^ vs. Yescarta^®^/Tecartus^®^ cohorts; (**E**) Comparison of CAR T-cell peak levels between all patients vs. Kymriah^®^ cohort vs. Yesarta^®^/Tecartus^®^ cohort; *Grey dashed line: Quartiles*; *Black dashed line: Median*; Abbreviations: K: Kymriah^®^; Mo: month(s); T: Tecartus^®^; Y: Yescarta^®^. Black: all patients; blue: patients treated with Kymriah^®^; green: patients treated with Yescarta^®^/Tecartus^®^.

Globally, CAR T-cell kinetics were similar between the two treatment cohorts (Kymriah^®^ vs. Yescarta^®^/Tecartus^®^). 96% of patients in the Kymriah^®^ cohort had still detectable CAR T-levels at 6 months, while only 65% of patients in the Yescarta^®^/Tecartus^®^ cohort were still positive by ddPCR (*p* = < 0.0001). In the first month post CAR T-cell treatment, the highest levels (median: 885 copies/μg cfDNA) and the widest variation of CAR T-cell copies was observed. CAR T-cell copies decreased progressively between month 3 and 6, stabilizing at approximately 100 copies/μg cfDNA afterwards (Figure 2A).

When comparing the two treatment cohorts, median copies/μg cfDNA were significantly higher at 1, 3, 6, 12, 18 and 24 months in the Kymriah^®^ cohort. Within the Yescarta^®^/Tecartus^®^ cohort, more patients showed negativized CAR T-cell copies at earlier time points (Figure 2B–D). Median CAR T-cell peak levels were similar between patients treated with Kymriah^®^ vs. Yescarta^®^/Tecartus^®^ (Figure 2E).

### 2.4. Treatment Outcome

Best responses following CAR T-cell therapy were complete response (CR) and PR in 47 (51%) and 23 (25%) patients, respectively. No significant differences were observed between patients with vs. without CAR T-cell persistence.

**Figure 3:**
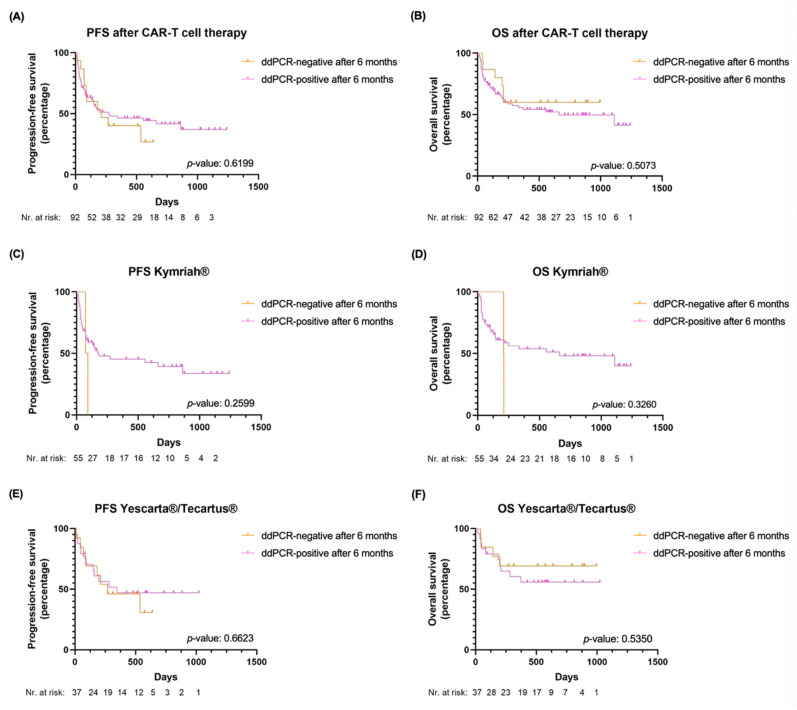
Clinical outcomes in patients with detectable vs. non-detectable CAR T-cells by ddPCR in the PB at 6 months post-CAR T-cell treatment (**A**) PFS in the entire cohort; (**B**) OS in the entire cohort; (**C**) PFS in patients treated with Kymriah^®^; (**D**) OS in patients treated with Kymriah^®^; (**E**) PFS in patients treated with Yescarta^®^/Tecartus^®^; (**F**) OS in patients treated with Yescarta^®^/Tecartus^®^. Abbreviations: OS: overall survival: PFS: progression-free survival. Orange: patients with negative ddPCR for CAR-T constructs in PB at 6 months; pink: patients with positive ddPCR for CAR-T constructs in PB at 6 months.

Median follow-up was 260 days (range: 3–1240). In total, 31 (34%) patients relapsed. While only 29% of relapses occured in the patient cohort with CAR T-cell persistence, 60% of patients without CAR T-cell persistence relapsed (*p* = 0.0336). 26 (28%) patients relapsed in the first 250 days (Figure 3A,C,E). A total of 42 (46%) patients died, most of them (38%) due to disease progression. Numerically more patients died within the CAR T-cell persistent cohort (47% vs. 40%, *p* = 0.7789). Median PFS was 9 (95% confidence interval (CI): 5-not reached (NR)) months and median OS 36 (95% CI: 8.2-NR) months. In the cohort of patients with CAR T-cell persistence, median PFS and OS were of 9 (95% CI: 5-NR) and 22 (95% CI: 8-NR) months, and in the patient population without CAR T-cell persistence 7 (95% CI: 3-NR) and NR (95% CI: 7-NR) months, respectively. After multivariable analysis, a trend towards longer PFS (HR 2.79, 95% CI: 1.09–7.11, *p* = 0.0319) and OS (HR 1.99, 95% CI: 0.68–5.82, *p* = 0.2092) was observed for patients with CAR T-cell persistence at 6 months (Table 4, Figure 3B,D,F). The 2 (100%) patients treated with Kymriah^®^ who negativized CAR T-cell ddPCR within the first 6 months died, whereas 49% with CAR T-cell persistence died (*p* = 0.4909). In the Yescarta^®^/Tecartus^®^ group the opposite trend was observed. (Figure 3, Supplementary Table S3).

## 3. Discussion

The emergence of CD19-targeting CAR T-cell therapies supposed a revolutionary advance in the treatment landscape of r/r B-cell malignancies [1,2,6,28,29,30,31,32]. However, tumors insufficiently responding or relapsing after CAR T-cell therapy still constitute a relevant challenge, and multiple aspects of tumor resistance to CAR T-cell therapies are incompletely understood [4,18,19]. Whether CAR T-cell long-term persistence is required to achieve sustained tumor responses is not yet fully clarified [33,34]. In this study, as an attempt to improve knowledge on this aspect, we analysed CAR T-cell pharmacokinetics assessed by ddPCR, and investigated the correlation of early CAR T-cell loss (<6 months) vs. persistence with treatment outcome.

A total of 92 B-cell lymphoma patients have been included in the study. We divided these patients into two cohorts: patients who negativized CAR T-cell ddPCR within the first 6 months (n = 15, 16%) vs. patients with CAR T-cell persistence (n = 77, 84%). Despite size difference, both cohorts were balanced as per basal patient and disease characteristics. The only statistically significant difference between the two cohorts was the initial lymphoma stage, with stage III being more frequent among patients with early CAR T-cell loss. No statistical differences for distribution of stage IV was observed.

We performed a second patient stratification based on the CAR T-cell product received (Kymriah^®^ vs. Yescarta^®^/Tecartus^®^). Kymriah^®^, Yescarta^®^ and Tecartus^®^ are three second generation CAR T-products, which are characterized by the presence of one costimulatory domain, either CD28 or 4-1BB, in addition to the CD3-ς signalling domain [16,35,36,37,38]. One relevant difference is that Kymriah’s CAR contains a 4-1BB-based co-stimulatory domain, and Yescarta^®^ and Tecartus^®^ a CD28-based [38]. While CD28-based CAR T-cells are characterized by a shorter persistence (about one month), the 4-1BB-based CAR T-cells typically persist for several years [39,40,41]. In our cohort we could confirm these findings. Only 13% of the patients who received Kymriah^®^ had a negative ddPCR result within the first 6 months. In contrast, 65% of patients treated with Yescarta^®^/Tecartus^®^ had no detectable circulating CAR T-cells at 6 months.

Globally, CAR T-cell kinetics were similar for the three CAR T-cell products, characterized by an initial expansion phase followed by the contraction and persistence phases. This finding correlates with the kinetics described in previous studies [10,42,43]. Remarkably, in our cohort, the median peak of CAR T-cell copies was significantly higher in patients with positive ddPCR at six months, as compared to patients who negativized within the first 6 months (5432 vs. 620 copies/μg cfDNA, *p* = 0.0096).

The overall response rate to CAR T-cell therapy was 76% in our cohort (51% CR, 25% PR). Most frequently best response was achieved within the first 3 months (86% of patients). A relapse was detected in 31 (34%) patients. Relapses were more frequent in patients with early CAR T-cell loss in the PB (60% vs. 29%, *p* = 0.0336). This finding aligns with other studies that suggest that persistence of CAR T-cells is associated with improved relapse-free survival [43].

Moreover, after performing multivariable analysis, we show that CAR T-cell persistence in the PB was associated with longer PFS (HR 2.79, *p* = 0.0319), whereas we found no significant differences for OS (HR: 1.99, *p* = 0.2092). In total, 46% of all patients died despite CAR T-cell treatment, most frequently due to disease progression (38%). There was no significant difference between the two patient cohorts (47% vs. 40%, *p* = 0.7789). This could be possible due to the relatively short follow-up of this study. In line with our findings, a study by Ayuk et al. showed that a higher AUC of axicabtagene ciloleucel correlated with better PFS [24]. Another approach to estimate persistence of functional CD19-targeting CAR T-cells is to monitor circulating levels of CD19-positive B-cells by flow cytoemtry. The absence of circulating CD19-positive B cells, also termed as B-cell aplasia, would suggest presence of active CD19-targeting CAR T-cells [44]. Further approaches to assess CAR T-cell expansion and activity include detection of upregulation of interleukin 15 (IL-15) and the granulocyte/macrophage colony-stimulating factor (GM-CSF) [45,46,47,48].

In our cohort, the incidence of ICANS was higher in patients with peripheral CAR T-cell persistence, while no differences in CRS and high-grade CRS frequency were observed. In previous studies, high level of CAR T-cell expansion, high tumor burden and occurrence of high-grade CRS were shown to be correlated with increased incidence of ICANS [42]. However, few data are available regarding CAR T-cell persistence.

Possible limitations of our study are the heterogeneity of our patient cohort, which included different histological subtypes (DLBCL, FL and MCL), and the unicentric study design. Additionally, longer-term follow-up is required to provide further insights into CAR T-cell kinetics and impact on treatment outcome.

## 4. Materials and Methods

### 4.1. Patients

We conducted a single-center retrospective observational study, analyzing data from r/r B-cell lymphoma patients treated with CAR T-cell therapy at the University Hospital of Bern, Switzerland, between 9th January 2019 and 1st October 2022. A total of 92 eligible patients diagnosed with r/r DLBCL, r/r MCL or r/r FL have been included in this study. For comparative analysis of clinical outcomes, we subdiveded the patients by CAR T-cell persistence at 6 months, and by administered CAR T-cell product. We retrospectively collected and analysed patient demographical and disease-related data. The study was conducted in accordance with the Declaration of Helsinki, as well as local laws and regulations.

### 4.2. Study Endpoints

Primary endpoint of the study was clinical outcome (relapse rate, PFS and OS) in patients with vs. without peripheral CAR T-cell persistence at 6 months. Secondary endpoints include correlation of CAR T-cell persistence and administered CAR T-cell product (Kymriah^®^ vs. Yescarta^®^/Tecartus^®^), as well as correlation with toxicities. CRS and ICANS clinical assessment and grading was performed following the American Society for Transplantation and Cellular Therapy (ASTCT) consensus grading.

### 4.3. Monitoring of CAR T-Cell Kinetics

CAR T-cell construct kinetics were monitored in PB using a previously established ddPCR assay [49]. This assay allows to quantify circulating copies of the intracellular junction domain located between the effector and co-stimulatory domains of the CAR [50]. Briefly, DNA was extracted from plasma samples using the QIAamp DNA minikit (Qiagen, Rotkreuz, Switzerland). The QX200 ddPCR system (Bio-Rad, Cressier, Switzerland) was used for the ddPCR assay. 250 ng of extracted DNA were used for the assay. DNA was digested with the restriction enzyme *Hae*III (New England Biolabs, Ipswich, MA, USA). The ddPCR reaction was prepared by combining the ddPCR Supermix for Probes (no dUTP, Bio-Rad, Cressier, Switzerland) with corresponding primers and probes. Ribonuclease P protein subunit 30 (RPP30) was taken as reference. Automated droplet generation was performed with the AutoDG droplet generator (Bio-Rad, Cressier, Switzerland), PCR was performed for 40 cycles (denaturation for 30 s at 94 °C, annealing and extension for 60 s at 55°). Number of CAR copies per μg of haploid DNA were calculated based on measurement of ribonuclease P protein subunit 30 (RPP30) concentrations, which allow to estimate the number of haploid genomes [50]. Primer design was performed in analogy to Milone et al. [51]. The limit of detection is 20 copies/μg of DNA. Patients with CAR T-construct concentration below this detection limit were considered as negative.

### 4.4. Statistical Analysis

PFS and OS curves were analyzed with the Kaplan-Meier method. PFS was defined as time from CAR T-cell treatment to any of the following events: relapse, death or lost to follow-up. OS was defined as time from CAR T-cell treatment to date of death.

GraphPad Prism 8^®^ was used for the graphical representation of figures and statistical analyses of tables and figures. Multivariable statistical analyses were performed with R^®^, version 4.1.2 (2021). Following variables have been included in multivariable analysis: age, sex, ddPCR negativity, DLBCL transformation, initial lymphoma stage, number of previous therapy lines, previous stem cell transplantation, bridging therapy, CAR T-cell product, remission status at CAR T-cell infusion, tocilizumab intake, steroids intake, LDH level before CAR T-cell therapy, CRS and ICANS. *p*-values below 0.05 were considered statistically significant and percentage results have been rounded to whole numbers. Cut-off regarding CAR T-cell infusion was 1 October 2022, cut-off concerning data completion was 11 October 2022.

## 5. Conclusions

Results from our study show that CAR T-cell persistence at 6 months correlates with improved clinical outcome in patients with B-cell lymphomas. Within the follow-up time period, we observed a lower rate of tumor relapses in patients with CAR T-cell persistence at 6 months. Moreover, a longer PFS and a non-significant trend towards improved OS was observed for this patient subgroup. Our data also confirms the previously reported longer persistence of 4-1BB vs. CD28-based CAR T-cells.

## Figures and Tables

**Table 1:** Baseline clinical characteristics of patients included in the study.

Parameter	All Patients	ddPCR-Negative at 6 Months	ddPCR-Positive at 6 Months	*p*-Value *
Patients	92 (100%)	15 (16%)	77 (84%)	>0.9999
Male/Female	56/36 (61/39%)	9/6 (60/40%)	47/30 (61/39%)	>0.9999
Median age at diagnosis, years (range)	62 (36–80)	62 (42–80)	63 (36–79)	0.4435
Histological subtype				
DLBCL	78 (85%)	13 (87%)	65 (84%)	0.344
Primary (de novo) DLBCL	52 (57%)	6 (40%)	46 (60%)	0.1108
Secondary DLBCL (transformed from)	26 (28%)	7 (47%)	19 (25%)	0.1108
FL	18 (20%)	5 (33%)	13 (17%)	>0.9999
CLL/SLL	5 (5%)	1 (7%)	4 (5%)	>0.9999
MZL	3 (3%)	1 (7%)	2 (2%)	>0.9999
Follicular lymphoma	2 (2%)	0 (0%)	2 (2%)	>0.9999
Mantle cell lymphoma	12 (13%)	2 (13%)	10 (13%)	>0.9999
Initial lymphoma stage (Ann Arbor)				
I	1 (1%)	0 (0%)	1 (1%)	>0.999
II	20 (22%)	2 (13%)	18 (20%)	0.5092
III	9 (10%)	5 (33%)	4 (5%)	0.0051
IV	43 (47%)	6 (40%)	37 (48%)	0.7782
Unknown	19 (21%)	2 (13%)	17 (22%)	0.7281
IPI score (for DLBCL)				
2	5 (5%)	0 (0%)	5 (6%)	0.5872
3	31 (34%)	7 (47%)	24 (31%)	0.2509
4 or 5	29 (32%)	4 (27%)	25 (32%)	0.7683
unknown	13 (14%)	2 (13%)	11 (14%)	>0.9999
MIPI (for Mantle cell lymphomas)				
4 to 5	5 (5%)	1 (7%)	4 (5%)	>0.9999
7	2 (2%)	0 (0%)	2 (2%)	>0.9999
9	1 (1%)	1 (7%)	0 (0%)	0.163
Unknown	4 (4%)	0 (0%)	4 (5%)	>0.9999
Hematopoietic stem cell transplantation before CAR T-cell therapy				
Autologous SCT	44 (48%)	9 (60%)	35 (45%)	0.3994
Allogeneic SCT	0 (0%)	0 (0%)	0 (0%)	>0.9999

CAR: chimeric antigen receptor; CLL: chronic lymphocytic leukaemia; ddPCR: droplet digital polymerase chain reaction; ddPCR negative: absence of CAR T-cell constructs by ddPCR; ddPCR positive: presence of CAR T-cell constructs by ddPCR; DLBCL: diffuse large B-cell lymphoma; IPI: International Prognostic Index; MIPI: Mantle Cell International Prognostic Index; MZL: marginal zone lymphoma; SCT: stem cell transplantation; SLL: small lymphocytic lymphoma. * Univariate analysis.

**Table 2:** CAR T-cell treatment and immune-related adverse events.

Parameter	All Patients n = 92 (100%)	ddPCR-Negative at 6 Months n = 15 (16%)	ddPCR-Positive at 6 Months n = 77 (84%)	*p*-Value *
Median age at CAR T-cell therapy, years (range)	68 (37–82)	65 (44–81)	69 (37–82)	0.6811
Number of treatment lines before CAR T-cell therapy				
1	0 (0%)	0 (0%)	0 (0%)	>0.9999
2	30 (33%)	2 (13%)	28 (36%)	0.1307
3	38 (41%)	9 (60%)	29 (38%)	0.1521
>3	24 (26%)	4 (27%)	20 (26%)	>0.9999
Previous radiotherapy	29 (32%)	4 (27%)	25 (32%)	0.7683
Remission status at the time of CAR T-cell therapy				
CR	5 (5%)	1 (7%)	4 (5%)	>0.9999
PR	34 (37%)	4 (27%)	30 (39%)	0.5598
SD	4 (4%)	1 (7%)	3 (4%)	0.5157
PD	49 (54%)	9 (60%)	40 (52%)	0.7782
Bridging therapy	47 (51%)	9 (60%)	38 (41%)	0.5753
Median LDH before lymphodepleting chemotherapy (U/l)	344 (126–3949)	295 (134–709)	351 (126–3949)	0.5843
Median time between lymphapheresis and CAR T-cell infusion, days (range)	48 (27–221)	42 (34–221)	48 (27–217)	0.2253
CAR T-cell product				
Kymriah^®^	55 (60%)	2 (13%)	53 (69%)	<0.0001
Yescarta^®^	25 (27%)	11 (73%)	14 (18%)	<0.0001
Tecartus^®^	12 (13%)	2 (13%)	10 (13%)	>0.9999
Cytokine-release syndrome (CRS)	73 (79%)	12 (80%)	61 (79%)	>0.9999
Grade 1	45 (49%)	9 (60%)	36 (47%)	0.4059
Grade 2	25 (27%)	2 (13%)	23 (30%)	0.3402
Grade 3	3 (3%)	1 (7%)	2 (3%)	0.4175
Grade 4	0 (0%)	0 (0%)	0 (0%)	>0.9999
Immune effector cell-associated neurotoxicity syndrome (ICANS)	30 (33%)	1 (7%)	29 (37%)	0.0182
Grade 1	8 (9%)	0 (0%)	8 (10%)	0.345
Grade 2	7 (8%)	0 (0%)	7 (9%)	0.594
Grade 3	10 (11%)	1 (7%)	9 (12%)	>0.9999
Grade 4	5 (5%)	0 (0%)	5 (6%)	0.5872
Median duration of hospitalization, days (range)	21.5 (5–73)	21 (18–42)	22 (5–73)	0.5631
Application of tocilizumab	59 (64%)	8 (53%)	51 (66%)	0.3852
Application of steroids	48 (52%)	5 (33%)	43 (56%)	0.1582

CR: complete response; LDH: lactate dehydrogenase; PD: progressive disease; PR: partial response; SD: stable disease; Tocilizumab: humanized monoclonal antibody against the interleukin-6 receptor. * Univaritate analysis.

**Table 3:** Parametres of CAR T-cell kinetics.

Parameter	Median CAR T-Cell Peak Level, Copies per µg cfDNA (Range)	Median Time between Day 0 and CAR T-Cell Peak Level, Days (Range)
All patients		
All patients n = 92 (100%)	4859.5 (37–127,942)	9 (2–83)
ddPCR-negative after 6 months n = 15 (16%)	620 (37–46,013)	12 (7–62)
ddPCR-positive after 6 months n = 77 (84%)	5432 (152–127,942)	9 (2–83)
*p*-value	0.0096	0.1006
Kymriah		
Total Kymriah n = 55 (100%)	4751 (320–127,942)	9 (2–37)
ddPCR-negative after 6 months n = 2 (4%)	23173 (333–46,013)	10 (9–11)
ddPCR-positive after 6 months n = 53 (96%)	4751 (320–127,942)	9 (2–37)
*p*-value	>0.9999	0.7205
Yescarta/Tecartus		
Total Yescarta/Tecartus n = 37 (100%)	5202 (37–92,877)	11 (7–83)
ddPCR-negative after 6 months n = 13 (35%)	620 (37–31,033)	13 (7–62)
ddPCR-positive after 6 months n = 24 (65%)	8297 (152–92,877)	10 (7–83)
*p*-value	0.0061	0.8723
Median time between day 0 and CAR-T peak level, days (range)		9 (2–83)
Median time between day 0 and negative ddPCR, days (range)		98 (17–651)
Median time between CAR T-cell peak level and negative ddPCR, days (range)		88 (10–642)
Median CAR-T cell level following CAR-T cell therapy, copies per µg cfDNA (range)		
	1 month	885 (0–93,100)
	3 months	145 (0–3871)
	6 months	94 (0–2666)
	12 months	110 (0–585)
	18 months	107.5 (0–1688)
	24 months	61 (0–423)
	30 months	78.5 (0–661)
	36 months	139 (0–469)

CfDNA: cell-free DNA; Day 0: CAR T-cell infusion day.

**Table 4:** Clinical outcome after CAR T-cell therapy.

Parameter	All Patients n = 92 (100%)	ddPCR-Negative at 6 Months n = 15 (16%)	ddPCR-Positive at 6 Months n = 77 (84%)	*p*-Value *
Best response				
CR	47 (51%)	8 (53%)	39 (51%)	>0.9999
PR	23 (25%)	5 (33%)	18 (23%)	0.5152
SD	7 (8%)	1 (7%)	6 (8%)	>0.9999
PD	15 (16%)	1 (7%)	14 (18%)	0.283
Time to best response, months				
0	24 (26%)	2 (13%)	22 (29%)	0.3378
1	31 (34%)	5 (33%)	26 (34%)	>0.9999
3	24 (26%)	7 (47%)	17 (22%)	0.0592
6	6 (7%)	0 (0%)	6 (8%)	0.5844
12	3 (3%)	0 (0%)	3 (4%)	>0.9999
15	1 (1%)	0 (0%)	1 (1%)	>0.9999
18	0 (0%)	0 (0%)	0 (0%)	>0.9999
21	1 (1%)	0 (0%)	1 (1%)	>0.9999
24	1 (1%)	1 (7%)	0 (0%)	0.163
27	1 (1%)	0 (0%)	1 (1%)	>0.9999
Response at last follow-up				
CR	42 (46%)	7 (47%)	35 (45%)	>0.9999
PR	16 (17%)	2 (13%)	14 (18%)	>0.9999
SD	6 (7%)	1 (7%)	5 (6%)	>0.9999
PD	28 (30%)	5 (33%)	23 (30%)	0.7676
Median follow up, days (range)	259.5 (3–1240)	313 (40–995)	224 (3–1240)	0.3683
Relapse,	31 (34%)	9 (60%)	22 (29%)	0.0336
Relapse treatment	26 (28%)	9 (60%)	17 (22%)	0.0092
Pharmacotherapy	22 (24%)	9 (60%)	13 (17%)	0.0011
Radiotherapy	12 (13%)	3 (20%)	9 (12%)	0.4063
Median time to relapse, days (range)	89.5 (11–863)	91 (11–536)	73 (12–863)	0.6828
Death, cause	42 (46%)	6 (40%)	36 (47%)	0.7789
disease related	35 (38%)	6 (40%)	29 (38%)	>0.9999
toxicity	1 (1%)	0 (0%)	1 (1%)	>0.9999
infection	2 (2%)	0 (0%)	2 (3%)	>0.9999
2nd malignancy	1 (1%)	0 (0%)	1 (1%)	>0.9999
other	3 (3%)	0 (0%)	3 (4%)	>0.9999
Median time to death, days (range)	81.5 (3–1110)	171.5 (40–210)	71 (3–1110)	0.3729
Comparison of OS			Mult. HR (95% CI)	*p*-value **
ddPCR-negative after 6 months			1.99 (0.68; 5.82)	0.2092
Comparison of PFS			Mult. HR (95% CI)	*p*-value **
ddPCR-negative after 6 months			2.79 (1.09; 7.11)	0.0319

* Univariate analysis; ** Multivariable analysis. Mult. HR: Multivariable hazard ratio (adjusted for age, sex, ddPCR negativity, DLBCL transformation, initial lymphoma stage, number of previous therapy lines, stem cell transplantation, bridging therapy, CAR T-cell product, remission status at CAR T-cell infusion, tocilizumab intake, steroids intake, LDH level before CAR T-cell therapy, CRS and ICANS).

## Data Availability

The datasets used and/or analysed during the current study are available from the corresponding author on reasonable request.

## Supplementary Materials

The following supporting information can be downloaded at: https://www.mdpi.com/article/10.3390/ijms24065688/s1.
