# Supplementary material for: CAR T-Cell Persistence Correlates with Improved Outcome in Patients with B-Cell Lymphoma
Source: Int J Mol Sci. 2023 Mar 16;24(6):5688. doi: 10.3390/ijms24065688 (PMC10056741; doi:10.3390/ijms24065688)
Supplement: Supplementary file 1 [file ijms-24-05688-s001.zip › ijms-2247333-supplementary.pdf]

CAR T-Cell Persistence Correlates with Improved Outcome in Patients with B-Cell Lymphoma

Original Study

Valerie Wittibschlager [1], Ulrike Bacher [2], Katja Seipel [1], Naomi Porret [2], Gertrud Wiedemann [2], Claudia Haslebacher [1], Michèle Hoffmann [1], Michael Daskalakis [2], Dilara Akhoundova[1] ‡, Thomas Pabst [1,*] ‡

‡ equal contribution

Affiliations: [1] Department of Medical Oncology; [2] Department of Hematology and Central Hematology Laboratory; Inselspital, University Hospital and University of Bern; Bern; Switzerland.

Supplemental Tables

Supplemental Table S1: Baseline clinical characteristics of patients included in the study stratified by received CAR T-cell product.

Parameter	Kymriah				Yescarta/Tecartus			
	**Total Kymriah®**	**ddPCR-negative at 6 months**	**ddPCR-positive at 6 months**	**P-value**	**Total Yescarta®/ Tecartus®**	**ddPCR-negative at 6 months**	**ddPCR-positive at 6 months**	**P-value**
**Patients**	55 (100%)	2 (4%)	53 (96%)	>0.9999	37 (100%)	13 (35%)	24 (65%)	>0.9999
**Demographic characteristics**								
**Male/Female**	33/22 (60/40%)	1/1 (50/50%)	32/21 (60/40%)	>0.9999	23/14 (62/38%)	8/5 (62/38%)	15/9 (63/37%)	>0.9999
**Median age at diagnosis, years (range)**	62 (37-79)	62.5 (62-63)	62 (37-79)	0.979	62 (36-80)	60 (42-80)	63.5 (36-78)	0.6529
**Diagnosis**								
DLBCL	53 (96%)	2 (100%)	51 (96%)	>0.9999	25 (68%)	11 (85%)	14 (58%)	0.1491
Primary (*de novo*) DLBCL	36 (65%)	1 (50%)	35 (66%)	0.5428	16 (43%)	5 (38%)	11 (46%)	0.1153
Secondary DLBCL, transformed from	17 (31%)	1 (50%)	16 (30%)	0.5428	9 (24%)	6 (46%)	3 (13%)	0.1153
Follicular lymphoma	12 (22%)	1 (50%)	11 (21%)	>0.9999	6 (16%)	4 (31%)	2 (8%)	>0.9999
CLL/SLL	3 (5%)	0 (0%)	3 (6%)	>0.9999	2 (5%)	1 (8%)	1 (4%)	>0.9999
MZL	2 (4%)	0 (0%)	2 (4%)	>0.9999	1 (3%)	1 (8%)	0 (0%)	>0.9999
Follicular lymphoma	2 (4%)	0 (0%)	2 (4%)	>0.9999	0 (0%)	0 (0%)	0 (0%)	>0.9999
Mantle cell lymphoma	0 (0%)	0 (0%)	0 (0%)	>0.9999	12 (32%)	2 (15%)	10 (42%)	0.1491
**Initial lymphoma stage**								
I	1 (2%)	0 (0%)	1 (2%)	>0.9999	0 (0%)	0 (0%)	0 (0%)	>0.9999
II	12 (22%)	1 (50%)	11 (21%)	0.3919	8 (22%)	1 (8%)	7 (29%)	0.2164
III	4 (7%)	0 (0%)	4 (8%)	>0.9999	5 (14%)	5 (38%)	0 (0%)	**0.003**
IV	26 (47%)	1 (50%)	25 (47%)	>0.9999	17 (46%)	5 (38%)	12 (50%)	0.7307
Unknown	12 (22%)	0 (0%)	12 (23%)	>0.9999	7 (19%)	2 (15%)	5 (17%)	>0.9999
**IPI score (DLBCL)**								
2	5 (9%)	0 (0%)	5 (9%)	>0.9999	0 (0%)	0 (0%)	0 (0%)	>0.9999
3	19 (35%)	1 (50%)	18 (34%)	>0.9999	12 (32%)	6 (46%)	6 (25%)	0.2738
4 or 5	21 (38%)	1 (50%)	20 (38%)	>0.9999	8 (22%)	3 (23%)	5 (17%)	>0.9999
unknown	8 (15%)	0 (0%)	8 (15%)	>0.9999	5 (14%)	2 (15%)	3 (13%)	>0.9999
**MIPI (Mantle cell lymphoma)**								
4 to 5	0 (0%)	0 (0%)	0 (0%)	>0.9999	5 (14%)	1 (8%)	4 (17%)	0.6378
7	0 (0%)	0 (0%)	0 (0%)	>0.9999	2 (5%)	0 (0%)	2 (8%)	0.5315
9	0 (0%)	0 (0%)	0 (0%)	>0.9999	1 (3%)	1 (8%)	0 (0%)	0.3514
Unknown	0 (0%)	0 (0%)	0 (0%)	>0.9999	4 (11%)	0 (0%)	4 (17%)	0.2756
**Hematopoietic stem cell transplantation before CAR-T cell therapy**								
Autologous SCT	26 (47%)	1 (50%)	25 (47%)	>0.9999	18 (49%)	8 (62%)	10 (42%)	0.3133
Allogeneic SCT	0 (0%)	0 (0%)	0 (0%)	>0.9999	0 (0%)	0 (0%)	0 (0%)	>0.9999

CLL: chronic lymphocytic leukaemia; DLBCL: diffuse large B-cell lymphoma; IPI: International Prognostic Index; MIPI: Mantle cell International Prognostic Index; MZL: marginal zone lymphoma; SCT: stem cell transplantation ; SLL: small lymphocytic lymphoma

Supplemental Table S2: Details of CAR T-cell treatment and immune-related adverse events for patients treated with Kymriah® vs. Yescarta®/Tecartus®.

Parameter		Kymriah®				Yescarta/Tecartus®			
		**Total Kymriah® n=55 (100%)**	**ddPCR-negative at 6 months n=2 (4%)**	**ddPCR-positive at 6 months n=53 (96%)**	**P-value**	**Total Yescarta®/ Tecartus® n=37 (100%)**	**ddPCR-negative at 6 months n=13 (35%)**	**ddPCR-positive at 6 months n=24 (65%)**	**P-value**
Median age at CAR T-cell therapy (range)		69 (38-82)	70.5 (69-72)	67 (38-82)	0.5418	64 (37-79)	64 (44-75)	70 (37-79)	0.675
**Number of treatment lines before CAR T-cell therapy**									
	1	0 (0%)	0 (0%)	0 (0%)	>0.9999	0 (0%)	0 (0%)	0 (0%)	>0.9999
	2	20 (37%)	0 (0%)	20 (38%)	0.5286	10 (27%)	2 (15%)	8 (33%)	0.4395
	3	26 (47%)	2 (100%)	24 (45%)	0.2189	12 (32%)	7 (54%)	5 (21%)	0.0666
	More than 3	9 (16%)	0 (0%)	9 (17%)	>0.9999	15 (41%)	4 (31%)	11 (46%)	0.4908
**Previous radiotherapy, yes**		17 (31%)	1 (50%)	16 (30%)	0.5266	12 (32%)	3 (23%)	9 (38%)	0.4757
**Remission status before CAR T-cell therapy**									
	CR	3 (5%)	0 (0%)	3 (6%)	>0.9999	2 (5%)	1 (8%)	1 (4%)	>0.9999
	PR	24 (44%)	2 (100%)	22 (42%)	0.1859	10 (27%)	2 (15%)	8 (33%)	0.4395
	SD	3 (5%)	0 (0%)	3 (6%)	>0.9999	1 (3%)	1 (8%)	0 (0%)	0.3514
	PD	25 (45%)	0 (0%)	25 (47%)	0.4949	24 (65%)	9 (69%)	15 (63%)	0.7343
**Bridging therapy, yes**		33 (60%)	1 (50%)	32 (60%)	>0.9999	15 (41%)	8 (62%)	7 (29%)	0.0829
**Median LDH before lymphodepleting chemotherapy (U/l)**		370 (145-3949)	353.5 (249-458)	370 (145-3949)	0.8047	257 (126-982)	295 (134-709)	217 (126-982)	0.5242
**Median duration between lymphapheresis and CAR T-cell infusion, days (range)**		60 (31-217)	66.5 (49-84)	60 (31-217)	>0.9999	40 (27-221)	41 (34-221)	38.5 (27-59)	0.4964
**Median duration of hospitalization, days (range)**		21 (5-51)	19.5 (19-20)	21 (5-51)	0.2411	25 (17-73)	22 (18-42)	27.5 (17-73)	0.0578
**Cytokine-release syndrome (CRS)**		45 (82%)	2 (100%)	43 (81%)	>0.9999	28 (76%)	10 (77%)	18 (75%)	>0.9999
	Grade 1	27 (49%)	2 (100%)	25 (47%)	0.2364	18 (49%)	7 (54%)	11 (46%)	0.7374
	Grade 2	16 (29%)	0 (0%)	16 (30%)	>0.9999	9 (24%)	2 (15%)	7 (29%)	0.4463
	Grade 3	2 (4%)	0 (0%)	2 (4%)	>0.9999	1 (3%)	1 (8%)	0 (0%)	0.3514
	Grade 4	0 (0%)	0 (0%)	0 (0%)	>0.9999	0 (0%)	0 (0%)	0 (0%)	>0.9999
**Immune effector cell-associated neurotoxicity syndrome (ICANS)**		17 (31%)	0 (0%)	17 (32%)	>0.9999	13 (35%)	1 (8%)	12 (50%)	**0.0129**
	Grade 1	4 (7%)	0 (0%)	4 (8%)	>0.9999	4 (11%)	0 (0%)	4 (17%)	0.2756
	Grade 2	6 (11%)	0 (0%)	6 (11%)	>0.9999	1 (3%)	0 (0%)	1 (4%)	>0.9999
	Grade 3	4 (7%)	0 (0%)	4 (8%)	>0.9999	6 (16%)	1 (8%)	5 (21%)	0.3945
	Grade 4	3 (6%)	0 (0%)	3 (6%)	>0.9999	2 (5%)	0 (0%)	2 (8%)	0.5315
**Use of tocilizumab**		33 (60%)	0 (0%)	33 (62%)	0.1556	26 (70%)	8 (62%)	18 (75%)	0.4645
**Use of steroids**		26 (47%)	0 (0%)	26 (49%)	0.4923	22 (59%)	5 (38%)	17 (71%)	0.0829

CR: complete remission; LDH: lactate dehydrogenase; PD: progressive disease; PR: partial remission; SD: stable disease

Supplemental Table S3: Clinical outcome after CAR T-cell therapy for patients treated with Kymriah® vs. Yescarta®/Tecartus®

Parameter		Kymriah®				Yescarta®/Tecartus®			
		**Total Kymriah® n=55 (100%)**	**ddPCR-negative at 6 months n=2 (4%)**	**ddPCR-positive at 6 months n=53 (96%)**	**P-value**	**Total Yescarta®/ Tecartus® n=37 (100%)**	**ddPCR-negative at 6 months n=13 (35%)**	**ddPCR-positive at 6 months n=24 (65%)**	**P-value**
**Best response**									
	CR	27 (49%)	1 (50%)	26 (49%)	>0.9999	20 (54%)	7 (54%)	13 (54%)	>0.9999
	PR	14 (25%)	1 (50%)	13 (25%)	0.4478	9 (24%)	4 (31%)	5 (21%)	0.711
	SD	4 (7%)	0 (0%)	4 (8%)	>0.9999	3 (8%)	1 (8%)	2 (8%)	>0.9999
	PD	10 (18%)	0 (0%)	10 (19%)	>0.9999	5 (14%)	1 (8%)	4 (17%)	0.6378
**Time to best response, months**									
	0	16 (29%)	0 (0%)	16 (30%)	>0.9999	8 (22%)	2 (15%)	6 (25%)	0.6851
	1	20 (36%)	2 (100%)	20 (38%)	0.1556	9 (24%)	3 (23%)	6 (25%)	>0.9999
	3	9 (16%)	0 (0%)	9 (17%)	>0.9999	15 (41%)	7 (54%)	8 (33%)	0.3003
	6	3 (5%)	0 (0%)	3 (6%)	>0.9999	3 (8%)	0 (0%)	3 (13%)	0.5382
	12	2 (4%)	0 (0%)	2 (4%)	>0.9999	1 (3%)	0 (0%)	1 (4%)	>0.9999
	15	1 (2%)	0 (0%)	1 (2%)	>0.9999	0 (0%)	0 (0%)	0 (0%)	>0.9999
	18	0 (0%)	0 (0%)	0 (0%)	>0.9999	0 (0%)	0 (0%)	0 (0%)	>0.9999
	21	1 (2%)	0 (0%)	1 (2%)	>0.9999	0 (0%)	0 (0%)	0 (0%)	>0.9999
	24	0 (0%)	0 (0%)	0 (0%)	>0.9999	1 (3%)	1 (8%)	0 (0%)	0.3514
	27	1 (2%)	0 (0%)	1 (2%)	>0.9999	0 (0%)	0 (0%)	0 (0%)	>0.9999
**Response at last follow-up**									
	CR	25 (45%)	0 (0%)	25 (47%)	0.4949	17 (46%)	7 (54%)	10 (42%)	0.5119
	PR	10 (18%)	0 (0%)	10 (19%)	>0.9999	6 (16%)	2 (15%)	4 (17%)	>0.9999
	SD	3 (5%)	0 (0%)	3 (6%)	>0.9999	3 (8%)	1 (8%)	2 (8%)	>0.9999
	PD	17 (31%)	2 (100%)	15 (28%)	0.0916	11 (30%)	3 (23%)	8 (33%)	0.7106
**Median follow-up, days (range)**		209 (3-1240)	209.5 (209-210)	174 (3-1240)	0.9791	425 (19-1023)	511 (40-271)	398.5 (19-1023)	0.4409
**Relapse**		18 (33%)	2 (100%)	16 (30%)	0.103	13 (35%)	7 (54%)	6 (25%)	0.1477
**Relapse treatment**		15 (27%)	2 (100%)	13 (25%)	0.0707	11 (30%)	7 (54%)	4 (17%)	**0.0275**
	Systemic treatment	12 (22%)	2 (100%)	10 (19%)	**0.0444**	10 (27%)	7 (54%)	3 (13%)	**0.0167**
	Radiotherapy	8 (15%)	1 (50%)	7 (13%)	0.2721	4 (11%)	2 (15%)	2 (8%)	0.6016
**Median time to relapse, days (range)**		72.5 (14-863)	81.5 (72-91)	71 (14-863)	0.7188	154.5 (11-536)	183 (11-536)	150 (12-347)	0.6026
**Death**		28 (51%)	2 (100%)	26 (49%)	0.4909	14 (38%)	4 (31%)	10 (42%)	0.7245
	Cause of death is disease related	23 (42%)	2 (100%)	21 (40%)	0.1704	12 (32%)	4 (31%)	8 (33%)	>0.9999
	Cause of death is toxicity	0 (0%)	0 (0%)	0 (0%)	>0.9999	1 (3%)	0 (0%)	1 (4%)	>0.9999
	Cause of death is an infection	2 (4%)	0 (0%)	2 (4%)	>0.9999	0 (0%)	0 (0%)	0 (0%)	>0.9999
	Cause of death is a 2nd malignancy	1 (2%)	0 (0%)	1 (2%)	>0.9999	0 (0%)	0 (0%)	0 (0%)	>0.9999
	Other cause	2 (4%)	0 (0%)	2 (4%)	>0.9999	1 (3%)	0 (0%)	1 (4%)	>0.9999
**Median time to death, days (range)**		71 (3-1110)	209.5 (209-210)	56.5 (3-1110)	0.2513	113 (19-372)	93 (40-201)	135 (19-372)	0.6354
